# Supplementary material for: Influence of Dynamic Strain Sweep on the Degradation Behavior of FeMnSi–Ag Shape Memory Alloys
Source: J Funct Biomater. 2023 Jul 19;14(7):377. doi: 10.3390/jfb14070377 (PMC10381450; doi:10.3390/jfb14070377)
Supplement: Supplementary file 1 [file jfb-14-00377-s001.zip › jfb-2457738-supplementary.pdf]

Supplementary data: *Influence of dynamic strain sweep on the degradation behavior of FeMnSi-Ag shape memory alloys*:

In Figure S1 are given the XRD spectra of the FeMnSi-2Ag at different temperatures (-50; 0; 25 and 100 °C) in order to confirm the presence of the main identified phases like γ, ε or α'.

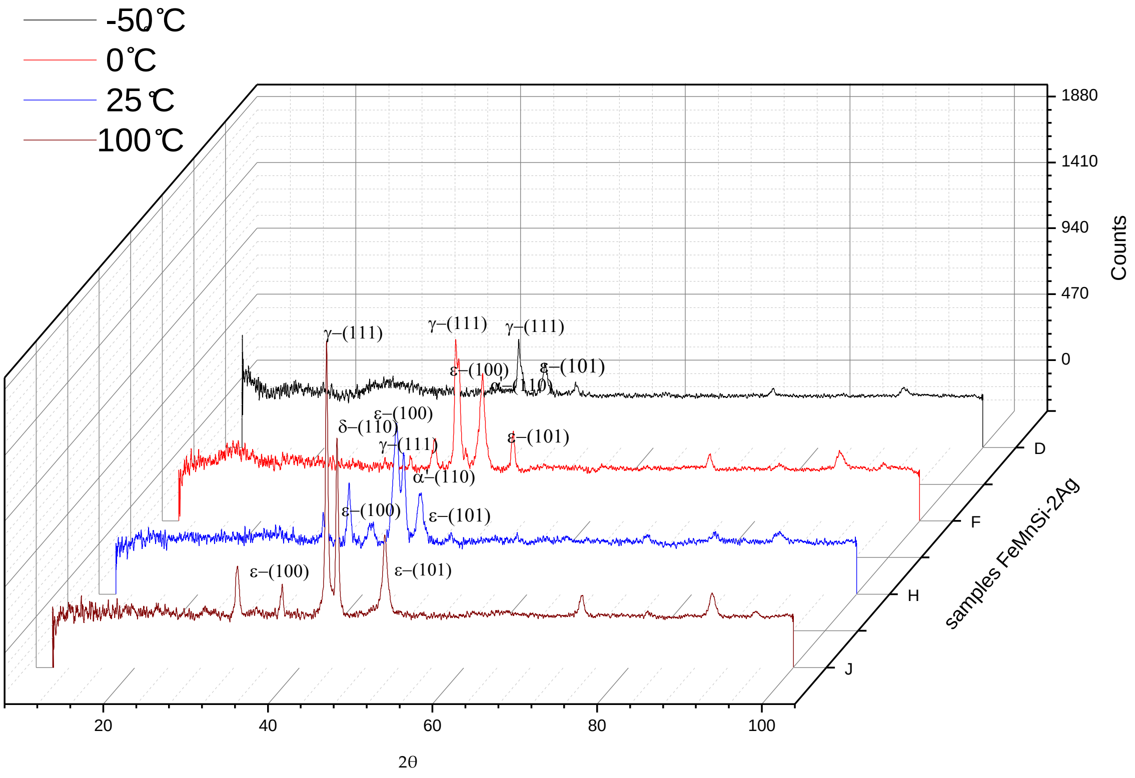

Figure S1 XRD patterns of the FeMnSi-2Ag sample -50°C, 0°C, 25°C, and 100°C

All samples analyzed are mainly austenitic phase type. The crystal structure of γ-austenite is face-centered-cubic (FCC), Copper-model - cF4. The cell parameter has a bigger value of the pure γ-Fe (0.36 nm), as preconized paying respect to the fact that this phase solid solution of Mn, Si and less Ag in Fe. Other common phases found in this system are δ-ferrite in the sample at 25 °C and stress-induced ε-martensite observed in all cases except the sample chiled down to -50 °C [59]. The δ-ferrite phase like type crystallizes in a body centered cubic - BCC structure, W-type - Im3m. On the other hand, ε-martensite exhibits hexagonal symmetry HCP, Mg-type -P63.

In this paper we focus more on the corrosion resistance and the alloy behavior in contact with Ringer solution and less on characterizing the shape memory effect of FeMnSi-

based alloys, subject extensively treated by other authors. FeMnSi-Ag lamella in hot-rolled state (relatively soft material with dimensions: 5x1x20 mm – Figure S2) after five cycles of deformation-heating steps (Figure S2) with a heating till 125 °C and cooling in water at room temperature present a partial recovery of the geometrical shape. The partial recovery of the shape was confirmed by visuals tests performed by deforming (22.5 º) and heating at 125 °C temperature, only for demonstration, the but no tensile experiments were performed at this moment.

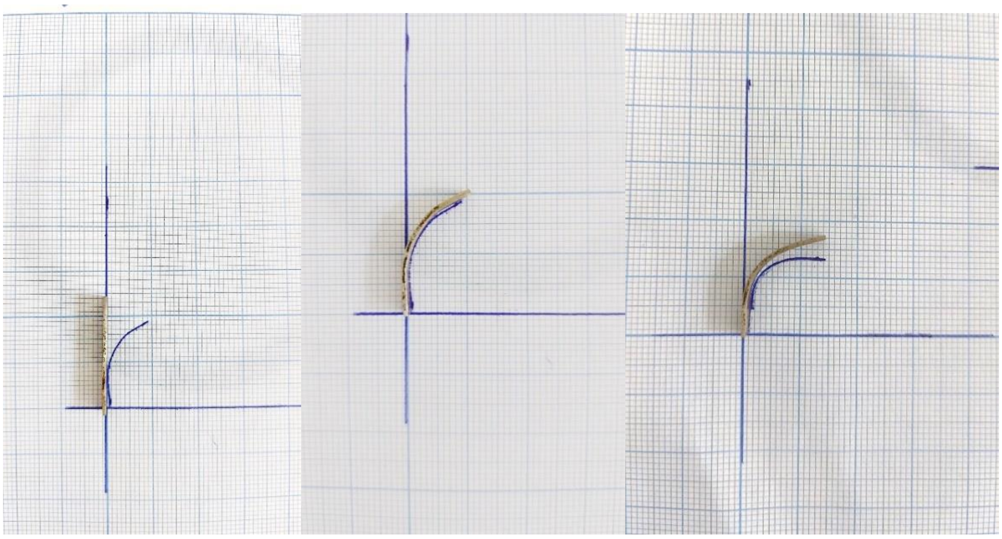

a) b) c)

Figure S2Partial recovery of the FeMnSi-Ag lamella after deformation and heating a) initial state, b) deformed and c) after heating

XRD results on the oxidized surface after immersion highlight the presence of γ and ε phases on the surface alongside with iron oxides, manganese oxide iron hydroxides and carbonates, Figure S3.

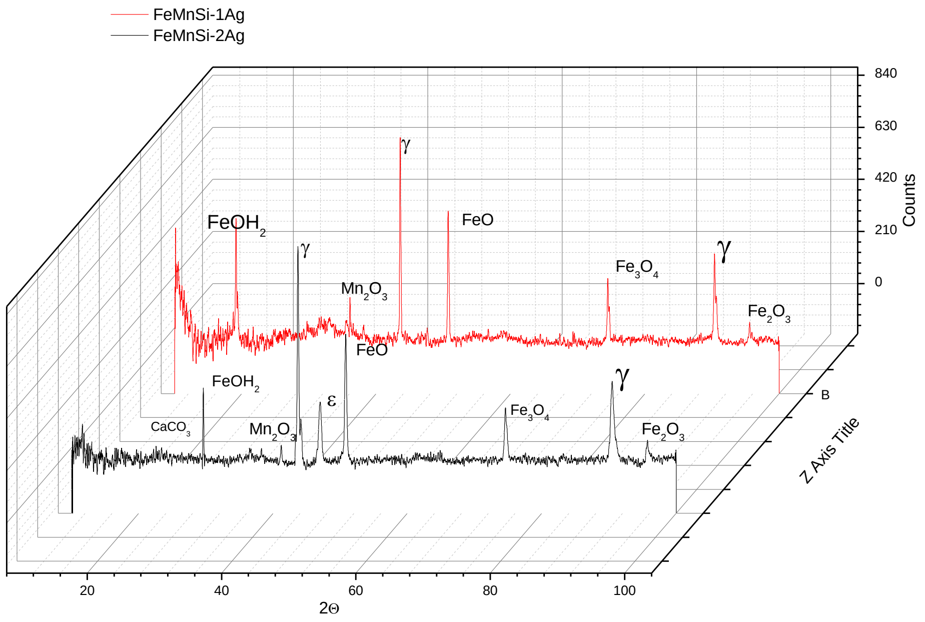

Figure S3XRD patterns of the oxidized surface of the FeMnSi-1(and 2) %Ag after 7 days of immersion in Ringer solution

XRD results on the oxidized surface after immersion highlight the presence of γ and ε phases on the surface alongside with iron oxides, manganese oxide, iron hydroxides and carbonates [71], Figure S3.
